# A review of the mechanisms underlying the role of the *GIPC3* gene in hereditary deafness

**DOI:** 10.3389/fnsyn.2022.1101587

**Published:** 2023-01-06

**Authors:** Xinxin Li, Lin Shi, Liang Wang

**Affiliations:** ^1^Department of Otolaryngology, First Affiliated Hospital of Dalian Medical University, Dalian, China; ^2^National Joint Engineering Laboratory, Stem Cell Clinical Research Center, Regenerative Medicine Center, First Affiliated Hospital of Dalian Medical University, Dalian, China

**Keywords:** *GIPC3*, auditory system, hereditary deafness, organ of Corti, gene mutations

## Abstract

The GAIP interacting protein c terminus (*GIPC*) genes encode a small family of proteins characterized by centrally located PDZ domains. *GIPC3* encodes a 312 amino acid protein. Variants of human *GIPC3* are associated with non-syndromic hearing loss. *GIPC3* is one of over a hundred different genes with variants causing human deafness. Screening for variants of *GIPC3* is essential for early detection of hearing loss in children and eventually treatment of deafness. Accordingly, this paper assesses the status of research developments on the role of *GIPC3* in hereditary deafness and the effects of pathogenic variants on the auditory system.

## 1. Introduction

Deafness is a major global public health issue and a common human disorder. According to the World Hearing Report, which was released by the World Health Organization (WHO), more than 1.5 billion people have varying degrees of hearing loss worldwide (Chadha et al., 2021; World Health Organization, 2021). Among these conditions of various degrees, sensorineural hearing loss (SNHL) is the most common congenital sensory disorder in children. The incidence of SNHL is one to two per thousand live births (van Beeck Calkoen et al., 2019), of which 70% are non-syndromic hearing loss (NSHL) (Sindura and Banerjee, 2019). Hereditary deafness is largely caused by gene mutations. More than 6,000 mutations in more than 150 genes have been causally linked to this condition, and *GJB2*, *SLC26A4*, *TMC1*, *OTOF*, and *CDH23* mutations account for the majority of these mutations. As research has progressed, it has been discovered that mutations in *GIPC3* play a significant role in the pathogenesis of various types of SNHL and are strongly associated with NSHL (Sloan-Heggen et al., 2016; Azaiez et al., 2018). However, many of the functions and pathogenic mechanisms of GIPC3 are still not fully elucidated. This review concludes the research developments on the involvement the *GIPC3* gene in hereditary deafness and its effects on the auditory system.

## 2. Overview of the *GIPC3* gene

### 2.1. Structure and function of the *GIPC3* gene

GIPC3 is a member of the GIPC family. The GAIP interacting protein, c terminus (GIPC), which interacts with the G Alpha-interacting protein (GAIP), is involved in the G protein-coupled signaling complex that constitutes the regulation of vesicle transport (De Vries et al., 1998). Saitoh et al. (2002) isolated *GIPC3* cDNAs from human fetal lung and discovered that *GIPC3* encodes a protein consisting of 312 amino acids with a central PDZ structural domain. Additionally, it shared 59.9% of its total amino acids with GIPC1, 55.3% of its total amino acids with GIPC2. These findings indicate that GIPC3 is highly correlated with GIPC1 and GIPC2. The *GIPC3* gene comprises six exons. It is located on chromosome 19 at region 19p13.3 and is associated with the thromboxane A2 receptor (*TBXA2R*) gene (Katoh, 2002). All GIPC family members contain the highly conserved GIPC homology 1 (GH1), GH2, and central PDZ domains, which interact with protein binding through their structural domains (Katoh, 2013).

### 2.2. Factors interacting with GIPC3

The GAIP interacting protein c terminus are widely involved in signal transduction, receptor and adhesion molecule transport, cell migration, and neurotransmitter release through protein interactions. They are also connected to numerous physiological and pathological processes in cells (Booth et al., 2002; Varsano et al., 2006; Yi et al., 2007; Prahst et al., 2008). The GH2 domain of GIPC directly interacts with the adaptor-binding domain 1 (ABD1) motif of myosin VI (MYO6) to oligomerize MYO6 and form the GIPC-MYO6 complex (Yu et al., 2009). Shang’s research analyzed the co-localization of Plexin D1 with GIPC1 and MYO6. It proved GIPC-mediated endocytosis and intracellular transport are important aspects of signal regulation of these receptors. Deafness caused by these mutations is therefore likely due to loss of the anchoring function of the GIPC3-MYO6 complex in stereocilia (Shang et al., 2017). The GIPC-MYO6 complex has been suggested to translocate early endosomes away from the plasma membrane through the cortical actin network and plays a pivotal role in the trafficking of transmembrane proteins on endocytic vesicles (Spudich et al., 2007; de Jonge et al., 2019). Myo6 is specifically expressed in mouse inner ear cochlear sensory hair cells, demonstrating that it is essential for hair cell formation and maintenance of their structural integrity (Avraham et al., 1995). Furthermore, it is required for effective Ca^2+^-dependent exocytosis in mature inner hair cell ribbon synapses (Roux et al., 2009). Myo6 and Gipc3 both have comparable localization and functions in mouse cochleae (Avraham et al., 1995; Charizopoulou et al., 2011). Therefore, this complex may be involved in signal transduction in the inner ear and may regulate Ca^2+^-dependent exocytosis at inner hair cell ribbon synapses (Roux et al., 2009). However, the exact mechanisms involved in this process are unclear. In addition, adaptor protein containing a PH domain and leucine zipper motif 1 (APPL1), which is a cytoplasmic signaling regulator, binds to the PDZ structural domain of GIPC3 protein and is crucial for GIPC3-mediated cell viability (Ding et al., 2017).

### 2.3. Localized expression study of *GIPC3*

By using Northern Blot analysis on human tissues, Saitoh et al. (2002) discovered that *GIPC3* mRNA was almost ubiquitously expressed in normal adult and normal fetal tissues. *GIPC3* mRNA was expressed at relatively higher level in the jejunum, followed by the lymph nodes, parietal lobes in brain, fetal spleen, and fetal thymus. However, *GIPC3* expression was not detected in the human ear at that time. As research progressed, Charizopoulou et al. (2011) used immunofluorescence staining to show that Gipc3 was exclusively localized in hair cells and spiral ganglion neurons (SGNs) of the mouse cochlea. In particular, distinct punctate staining was observed at the base of inner hair cells (IHCs) and outer hair cells (OHCs), around the nucleus, and throughout the apical region of the hair cells. These findings also laid the foundation for the subsequent finding that *GIPC3* mutations trigger hearing loss in human and mouse. Abundant staining was also detected in the cytoplasm of vestibular hair cells. However, there was no research in potential role of *GIPC3* in genetic vestibular disorders.

## 3. Effects of *GIPC3* mutations on the auditory system

### 3.1. Several common *GIPC3* mutations that trigger hereditary non-syndromic deafness

*GIPC3* is commonly expressed in adult tissues, and *GIPC3* mutations have an impact on several auditory system components. In humans, *GIPC3* mutations can lead to NSHL, a disorder in which hearing loss is caused by damage to the inner ear, the auditory nerve, or the auditory center of the brain. NSHL manifests only as abnormalities of the auditory system without lesions in other organs and systems. It demonstrates significant locus and allelic heterogeneity and is inherited in a variety of ways (Gao et al., 2017). For 80% of NSHL cases, the mode of inheritance is autosomal recessive. To better understand the specific locus of the *GIPC3* mutations that cause NSHL, Chen et al. (1997) applied gene sequencing to map a new locus of autosomal recessive non-syndromic hearing loss (ARNSHL), and it mapped to *DFNB15* on chromosome 19p. In a Dutch ARNSHL family, Charizopoulou et al. (2011) identified a nucleotide substitution in exon 6 that localized to *DFNB95* on chromosome 19p by *GIPC3* mutation analysis. Through sequencing analysis, a missense mutation in exon 5 located at *DFNB15* on chromosome 19p was identified in an Indian ARNSHL family. Six missense mutations and one shift mutation in *GIPC3*, located at *DFNB72* on chromosome 19p, were found in seven Pakistani families with varying degrees of sensorineural deafness (Ain et al., 2007; Rehman et al., 2011). In summary, *GIPC3* mutations have been shown to underlie NSHL associated with *DFNB15*, *DFNB72* and *DFNB95*. In NSHL, biallelic, nonsense, and shift mutations were also observed in the *GIPC3* gene (Chen et al., 1997; Ain et al., 2007; Charizopoulou et al., 2011; Rehman et al., 2011; Diaz-Horta et al., 2012; Sirmaci et al., 2012; Ramzan et al., 2013; Petrova et al., 2021), and more *GIPC3* mutation loci are gradually being reported as research progresses (Siddiqi et al., 2014; Ammar-Khodja et al., 2015; Asgharzade et al., 2018; Khan et al., 2019; Bitarafan et al., 2020; Kannan-Sundhari et al., 2020; Zhou et al., 2020; Table 1).

**TABLE 1:** Summaries *GIPC3* mutations associated with autosomal recessive non-syndromic hearing impairment.

Mutation	Mutation type	Protein change	Domain	Population	Hearing loss phenotype	References
c.122C > A	Missense	p. Thr41Lys	GH1	Saudi Iranian	Severe to profound	Ramzan et al., 2013; Bitarafan et al., 2020
c.136G > A	Missense	p. Gly46Arg	GH1	Pakistan	Severe to profound	Rehman et al., 2011
c.226-1G > T	Canonical splice site	p. Ile76ProfsX34	GH1	Pakistan	Severe to profound	Siddiqi et al., 2014
c.245A > G	Missense	p. Asn82Ser	GH1	Chuvash Iranian	Severe to profound	Bitarafan et al., 2020; Kannan-Sundhari et al., 2020; Petrova et al., 2021
c.264G > A	Missense	p. Met88Ile	GH1	Pakistan	Mild to severe	Rehman et al., 2011
c.281G > A	Missense	p. Gly94Asp	GH1	Pakistan	Mild to severe	Rehman et al., 2011
c.472G > A	Missense	p. Glu158Lys	PDZ	Iranian	Severe to profound	Asgharzade et al., 2018
c.508C > A	Missense	p. His170Asn	PDZ	Turkish	Severe to profound	Diaz-Horta et al., 2012; Sirmaci et al., 2012
c.565C > T	Missense	p. Arg189Cys	PDZ	Pakistan	Severe to profound	Rehman et al., 2011
c.662C > T	Missense	p. Thr221Ile	GH2	Pakistan	Profound	Rehman et al., 2011
c.685dupG	Frameshift	p. Ala229GlyfsX10	GH2	Pakistan	Moderate to severe	Rehman et al., 2011
c.759C > G	Missense	p. Ser253Arg	GH2	Pakistan	Severe to profound	Khan et al., 2019
c.764T > A	Missense	p. Met255Lys	GH2	Algeria	profound	Ammar-Khodja et al., 2015
c.767G > A	Missense	p. Gly256Asp	GH2	Pakistan	Moderate to severe	Rehman et al., 2011
c.785T > G	Missense	p. Leu262Arg	GH2	Indian	Severe to profound	Charizopoulou et al., 2011
c.903G > A	Non-sense	p. Trp301X	GH2	Dutch	Severe to profound	Charizopoulou et al., 2011
c.937T > C	Stop codon lost	p.*313Gluext*98	GH2	Pakistan	Severe to profound	Zhou et al., 2020
c.343G > A	Missense	p. Gly115Arg	PDZ	BLSW mice	age-related hearing loss	Charizopoulou et al., 2011

### 3.2. Effect of *Gipc3* mutations on mechanical conduction in the inner ear

Mechanical conduction and electrical signal conduction coexist in the inner ear, and the integrity of both is critical to sound perception. Each part of the cochlea is involved in this process. Mechanical conduction in the peripheral auditory system depends on the movement of hair cells and their stereocilia. Sound transmission into the inner ear causes vibrations in the basilar membrane, which causes shearing movements in the reticular layer of the organ of Corti and the tectorial membrane. This progress causes the bending of the OHC stereocilia and opening of the ion channels. This induces the amplification of sound-evoked vibrations that reach the cochlea and are transmitted to the IHC. These effects further give rise to neurotransmitter release, generating electrical signals that pass from the SGNs to the superior olivary nucleus complex. Then, the signals are transmitted to the lateral thalamus and inferior colliculus. Finally, they are passed through the medial geniculate afferent fibers, terminating in the auditory cortex of the brain to generate auditory sensation (Ren, 2005).

**FIGURE 1:**
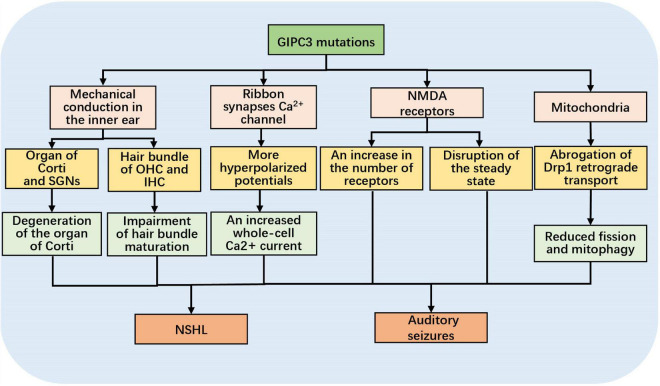
Schematic diagram of the mechanisms of hearing loss and acoustic seizures triggered by *GIPC3* mutations.

Charizopoulou et al. (2011) identified that the progressive sensorineural deafness (age-related hearing loss 5, *ahl5*) and audiogenic seizures (juvenile audiogenic monogenic seizure 1, *jams1*) that develop in Black Swiss (BLSW) mice were associated with 343G > A mutation in the PDZ structural domain of *Gipc3*. They also discovered that, in contrast to C3HeB/FeJ mice, BLSW mice prematurely exhibited OHC and IHC stereocilia bundle defects, such as stereocilia sparseness, stereocilia disorder, and impaired maturation. Additionally, with age progression, there was marked degeneration of the organ of Corti, a loss of SGNs, and reduced efficiency in OHC function, as revealed by distortion product otoacoustic emissions (DPOAEs) and lower levels of Gipc3 protein expression in afferent neurons of hair cells and SGNs. Thus, Gipc3 is necessary for postnatal stereocilia maturation and the long-term survival of hair cells and SGNs. When *Gipc3* is mutated, it causes mechanical conduction disorders in the inner ear (Figure 1).

### 3.3. Effect of *Gipc3* mutations on Ca^2+^ influx and spiral ganglion cells

Sound signals are converted into electrical signals in hair cells, which are transmitted *via* auditory nerve fibers to spiral ganglion cells and then to all levels of centers. The process of mechanical-electrical conversion in hair cells is achieved *via* synapses. The ribbon synapse serves as the critical location for exocytosis; the neurotransmitter glutamate is released in response to mechanical stimuli. In contrast, transmitter release from cochlear hair cells is triggered by Ca^2+^ influx through the dihydropyridine-sensitive L-type voltage-gated calcium channel (VGCC) (Fuchs et al., 2003; Frank et al., 2009). Inner hair cell ribbon synapses contain a host of active zones (AZs), and each AZ houses multiple Ca^2+^ channels. Ohn et al. (2016) found that the number of Ca^2+^ channels varied among AZs and that larger, more complicated AZs had more Ca^2+^ channels, creating a modiolar-pillar gradient at the inner hair cell ribbon synapses. On the side near the SGNs, the larger AZ has more Ca^2+^ channels and is able to associate with SGNs with lower spontaneous firing frequencies and higher auditory nerve firing thresholds. Conversely, on the side away from the SGNs, AZs can be associated with SGNs with higher spontaneous firing frequencies and lower auditory nerve firing thresholds. However, the voltage dependence of Ca^2+^ influx varied with AZ location, showing a gradient opposite to that of maximal Ca^2+^ influx. In the IHC of BLSW mice, disruption of *Gipc3* reversed the modiolar-pillar gradient of maximal AZ Ca^2+^ influx, shifting the activation voltage of Ca^2+^ channels to a more hyperpolarized potential. This, in turn, caused an increase in IHC whole-cell Ca^2+^ influx and increased the proportion of SGNs with high spontaneous discharge rates (Figure 1). For this reason, *Gipc3* mutations may therefore affect the hyperpolarization potential of Ca^2+^ channels and alter AZ gradients.

### 3.4. Association between *Gipc3* mutations and auditory seizures

Audiogenic seizures are a panic episode brought on by sudden high-intensity noise. Auditory seizures in BLSW mice are characterized by uncontrolled running, loss of the righting reflex, and tonic flexion and extension. This seizure susceptibility is developmentally regulated. It peaks at 2–3 weeks of age and almost completely vanishes by adulthood (Misawa et al., 2002). Charizopoulou et al. (2011) found that BLSW mice were most susceptible to seizures at 2–4 weeks of age, and the latency for auditory seizures was significantly shorter than that of C3HeB/FeJ mice in the control group. The seizure latency increased significantly between 3 and 5 weeks of age. However, full tolerance occurred at 6 weeks of age, with no significant difference in latency duration compared to controls. The ABR I-wave amplitude represents the total activity of cochlear nerve fibers projecting from the IHC to the cochlear nucleus. The ABR I-wave amplitude was significantly higher in 2-week-old BLSW mice than in control C3HeB/FEJ mice under 100 dB SPL sound stimulation and that these high amplitudes decreased rapidly at 7 weeks of age. A similar pattern of changes in ABR wave amplitude and seizure latency were also observed. Therefore, it was concluded that the ABR I-wave amplitude decreased after a significant increase, which was associated with epilepsy susceptibility and resistance, respectively. Additionally, the quantitative trait locus (QTL) of *jams1* was determined to be located within the critical interval of the QTL of *ah15*. Furthermore, the *Gipc3* transgene could compensate for the hearing defect caused by *ah15* in *Gipc3*^343*A/A*^ homozygotes and attenuate the susceptibility to auditory convulsions in *jams1* by prolonging the latency of auditory seizures. Therefore, there is a direct link between mutations in *Gipc3* and the onset and development of auditory seizures in mouse.

There is a lack of relevant reports on the mechanism of acoustic seizure in *Gipc3* mutant mice, previous researches hypothesized there could be two causes: 1. cochlear nerve fibers became high spontaneous discharge due to an increase in IHC whole-cell Ca^2 +^ influx (see part 3.3). 2 imbalance of NMDA receptors caused by *Gipc3* mutation: In the cochlea, glutamate receptors mediate rapid IHC-related synaptic transmission and glutamate excitotoxicity, which is the key point of nerve damage during auditory trauma (Puel, 1995). Two types of glutamate receptors have been confirmed in the auditory transmission pathway, ionotropic receptors, known as AMPA receptors, and metabotropic receptors, known as NMDA receptors. NMDA receptors are found in the majority of glutamatergic synapses in the mammalian central nervous system and are essential for synaptic transmission, synaptic plasticity, and synaptic development. It has been shown that GIPC is widely expressed in neurons, both pre- and postsynapses (Jeanneteau et al., 2004). GIPC binds to NMDA receptors and modifies the amount of NMDA receptors. According to research by Yi et al. (2007) GIPC also acts as an antagonist, preventing NMDA receptors from interacting with other PDZ proteins. A crucial mechanism for modulating glutamate receptor function is controlling the amount of cell surface receptors. As a member of the GIPC family, *Gipc3* mutations may result in an increase in the number of NMDA receptors and disruption of the steady state, both of which lead to auditory seizures in mouse (Figure 1). However, it is unknown why acoustic seizures are only seen in animals with the *Gipc3* mutation and why they stop occurring at six weeks after birth.

### 3.5. Effect of *GIPC3* mutations on mitophagy

Mitochondria are the primary site of energy production by aerobic respiration in which, catalyzing the phosphorylation of intracellular ADP to ATP and providing energy. Mitochondria are crucial for maintaining the dynamic equilibrium of cellular redox, signal transduction, and the regulation of apoptosis (Chen and Tang, 2014). Mitophagy is a process that selectively isolates and degrades damaged mitochondria. It is a specific quality control mechanism that plays an important role in maintaining mitochondrial function, integrity, and dynamic homeostasis (Youn et al., 2020). Impaired mitophagy results in the progressive accumulation of defective mitochondria, leading to cell and tissue damage (Dombi et al., 2018). It has been found that the induction of mitophagy attenuates the cytotoxicity of carbonyl cyanide m-chlorophenylhydrazone (CCCP) on auditory cells, thereby protecting the auditory system (Setz et al., 2018). In addition, it has also been established that the upregulation of mitophagy can minimize damage to the inner ear and prevent hearing loss (Ye et al., 2018).

The process of mitophagy requires the synergy of multiple factors. Lin et al. (2019) demonstrated that Dynamin-related Protein 1 (Drp1) plays a central role in mitophagy by regulating mitochondrial fission. Inhibition of Drp1 promotes cochlear hair cell senescence and exacerbates age-related hearing loss. Ramonett et al. (2022) demonstrated the interaction between GIPC and Drp1 by immunoprecipitation, among other methods, and found that Drp1 interacts with GIPC through its atypical C-terminal PDZ-binding motif. GIPC directs the aggregation of Drp1 in perinuclear mitochondria, which in turn promotes mitochondrial fission; thus, GIPC-mediated Drp1 retrograde plays a key role in mitochondrial fission. Previous research has demonstrated that the interaction between GIPC and Drp1 promotes mitochondrial fission and thus affects the mitophagy process (Figure 1). Therefore, when *GIPC3* is mutated, the interaction between the two may be impacted. This allows for the inhibition of Drp1 aggregation in perinuclear mitochondria and reduced mitophagy, contributing to NSHL.

## 4. Conclusion and outlook

GIPC3, as a member of the GIPC family, has important implications in physiological and pathological processes, such as signal transduction, substance transport, cell migration, and neurotransmitter release. *GIPC3* has been found to be most closely associated with hereditary deafness, and *GIPC3* mutations are the foundation of *DFNB15*, *DFNB72*, and *DFNB95*-associated NSHL.

Several mechanisms by which *GIPC3* mutations may cause NSHL and acoustic seizures have been suggested. First, Gipc3 is required for postnatal stereocilia maturation and the long-term survival of hair cells and SGNs. When *Gipc3* is mutated, the reduced Gipc3 protein expression compromises the inner ear structures, which are used for mechanotransduction and signal amplification. Second, the *Gipc3* mutations shift the activation voltage of Ca^2+^ influx to a more hyperpolarized potential and reverse the modiolar-pillar gradient of maximal Ca^2+^ influx while increasing the proportion of SGNs with high spontaneous discharge rates. Third, GIPC can adjust the number of NMDA receptors by binding to NMDA receptors, and GIPC can prevent NMDA receptors from interacting with other PDZ proteins, acting as an antagonist. *GIPC3* mutations may increase the number of NMDA receptors and disrupt homeostasis, which can result in auditory seizures. Fourth, Drp1 interacts with GIPC through a C-terminal PDZ-binding motif. GIPC directs the aggregation of Drp1 in perinuclear mitochondria, promoting mitophagy. When *GIPC3* is mutated, the interaction between the two may be inhibited, which in turn leads to NSHL.

It is unclear how *GIPC3* mutations cause sensorineural deafness and acoustic seizures. For example, it is unclear how *GIPC3* mutations trigger impaired stereocilia maturation, whether *GIPC3* mutations inhibit Drp1-mediated mitophagy, and whether it triggers cochlear autoimmune reactions. To better understand the molecular biological mechanisms underlying genetic deafness due to *GIPC3* mutations and to provide interventions, further experimental validation is needed. In conclusion, *GIPC3* is a scientifically valuable focus in the study of the developmental mechanisms of hereditary deafness.

## Author contributions

XL consulted the literatures and wrote the manuscript. LS and LW participated in drafting of the manuscript and provided oversight for the project. LS was responsible for conducting manuscript evaluation. All authors read and approved the final manuscript.
